# Probucol prevents atrial ion channel remodeling in an alloxan-induced diabetes rabbit model

**DOI:** 10.18632/oncotarget.13339

**Published:** 2016-11-14

**Authors:** Huaying Fu, Guangping Li, Changle Liu, Jian Li, Lijun Cheng, Wansong Yang, Gary Tse, Jichao Zhao, Tong Liu

**Affiliations:** ^1^ Department of Cardiology, Tianjin Key Laboratory of Ionic-Molecular Function of Cardiovascular disease, Tianjin Institute of Cardiology, Second Hospital of Tianjin Medical University, Tianjin, People's Republic of China; ^2^ Department of Medicine and Therapeutics, Chinese University of Hong Kong, Hong Kong, SAR, P.R. China; ^3^ Li Ka Shing Institute of Health Sciences, Chinese University of Hong Kong, Hong Kong, SAR, P.R. China; ^4^ Auckland Bioengineering Institute, The University of Auckland, New Zealand

**Keywords:** diabetes mellitus, atrial fibrillation, alloxan-induced diabetes, atrial ionic remodeling, calcium current, Pathology Section

## Abstract

Diabetes mellitus (DM) increases the risk of developing atrial fibrillation (AF), but the molecular mechanisms of diabetes-induced atrial remodeling processes have not been fully characterized. The aim of this study was to examine the mechanisms underlying atrial ion channel remodeling in alloxan-induced diabetes model in rabbits. A total of 40 Japanese rabbits were randomly assigned to a control group (C), alloxan-induced diabetic group (DM), probucol-treated control group (Control-P), and probucol-treated diabetic group (DM-P). Using whole-cell voltage-clamp techniques, *I*_Ca,L_, *I*_Na_ and action potential durations (APDs) were measured in cardiomyocytes isolated from the left atria in the four groups, respectively. In the DM group, increased *I*_ca,L_ and decreased *I*_Na_ currents were reflected in prolonged APD_90_ and APD_50_ values. These changes were reversed in the DM-P group. In conclusion, probucol cured AF by alleviating the ion channel remodeling of atrial myocytes in the setting of diabetes and the promising therapeutic potential of anti-oxidative compounds in the treatment of AF warrants further study.

## INTRODUCTION

Nowadays, atrial fibrillation (AF) is the most common arrhythmic condition encountered in clinical practice, producing significant morbidity and mortality via the development of various concurrent diseases and consequences, such as strokes and myocardial infarction [1]. Unfortunately, its prevalence and incidence continue to accelerate, placing a significant financial burden on the healthcare systems worldwide. Current available clinical treatment strategies, such as pharmacological therapy or invasive procedures such as catheter ablation have limited efficacy and are not without adverse effects, especially for patients with persistent AF. As one independent risk factor of AF, diabetes mellitus (DM) is a clinical condition that is becoming increasingly prevalent, in part due to a rising level of obesity, and has been shown to increase atrial arrhythmogenicity through structural and electrophysiological remodeling [2-4]. The exact underlying pathophysiological mechanisms of such remodeling processes are not fully understood [5]. In an alloxan-induced diabetic rabbit model, our group had previously demonstrated that hyperglycemic conditions lead to increased interstitial fibrosis and higher likelihood of AF development[7], as well as reduction of the Na^+^ current (*I*_Na_) and increasement of the Ca^2+^ current (*I*_CaL_) in the atria [8]. In the past, it had been demonstrated that an increase in oxidative stress leads to shortened action potential durations (APDs) in the atria due to the elevated transient outward K^+^ current [10] and promotes Ca^2+^ release from the intracellular store [11]. Furthermore, reduction of I_Na_ current due to reduced mRNA and protein levels was also observed [12]. Together, these changes can lead to both triggered and re-entrant arrhythmogenesis.

Probucol is an anti-hyperlipidemic medication with antioxidant effects by reducing lipid peroxidation and increasing the activities of antioxidant enzymes. Our previous study has demonstrated that probucol exerted anti-inflammatory effects in the atria, through downregulation of a number of key genes involved in the pro-inflammatory pathways, such as NF-κB, TGF-β, HSP70, TNF-α in DM rabbit models [9]. However, electrical remodeling under DM and the impact of probucol have not been studied and reported. In this present study, by using an integrative approach of molecular biology and electrophysiology techniques on well established animal models, we tested the hypothesis that probucol can prevent ion channel remodeling in the atria by its anti-inflammatory and anti-oxidative impact.

## RESULTS

### Serum oxidative stress parameters

The levels of biochemical and oxidative stress markers in the four experimental groups are illustrated in Table 1. We discovered that DM led to increased levels of cholesterol, triglyceride and LDL-c, as well as higher MPO, and lower SOD and CAT concentrations compared to control (*P* < 0.05), but not significance in renal function (BUN and creatinine values) (*P* > 0.05). The probucol treatment reversed the rise in the lipid peroxidation product, malondialdehyde (MDA) in the DM group (*P* < 0.05).

**Table 1:** Biochemical and oxidative stress parameters

	Control group (*n* = 10)	DM group (*n* = 10)	DM-P group (*n* = 10)	control-P group (*n* = 10)
Weight, Kg	2.19±0.17	2.29±0.47	2.29±0.20	2.34±0.08
Glu level at 8W,mmol/L	5.58± 0.75	20.86±4.37*	19.28±8.82*	6.54±0.52#
BUN, mmol/L	5.65±2.00	6.87±2.23	5.08±1.01	4.49±1.40
Cr, umol/L	98.5±16.31	103.7±29.22	95.13±27.18	89.66±9.48
TG, mmol/L	1.19±0.31	1.52±1.23	1.30±0.29	1.25±0.38
TC, mmol/L	1.14±0.57	1.25±0.39	0.87±0.35	0.92±0.36
HDL-c, mmol/L	0.56±0.16	0.49±0.20	0.43±0.14	0.47±0.17
LDL-c, mmol/L	0.36±0.16	0.38±0.16	0.31±0.15	0.24±0.10
INS, uIU/ml	16.24±7.87	5.71±3.01*	7.33±1.56*	13.89±5.11#
Serum CAT, U/ml	6.40±2.46	4.79±2.06	4.80±0.96	5.35±1.74
Serum SOD, U/ml	588.08±82.09	556.29±47.7	589.18±63.44	640.12±82.02
Serum MPO, U/L	63.66±29.44	76.41±27.02	56.66±20.58	64.30±14.35
Serum MDA, nmol/ml	17.69±3.59	22.17±4.15*	18.32±1.63#	18.14±2.62#
CAT in atrium, U/mg prot	4.30±1.29	3.82±1.87	4.00±1.13	4.78±1.33
SOD in atrium, U/mg prot	98.41±12.07	72.11± 11.08*	88.48±8.81#	93.03±20.04#
MPO in atrium, U/mg prot	0.51±0.13	1.00±0.38*	0.78±0.08*	0.55±0.91#
MDA in atrium, mol/mg prot	0.78±0.12	1.10±0.24*	0.84±0.32#	0.64±0.17#

Values are mean ± SD; Abbreviations: LA-left atrial; LV-left ventricular;

* Compared with Control group *p* < 0.05;

# Compared with DM group *p* < 0.05

### I_Na_ densities

**Figure 1:**
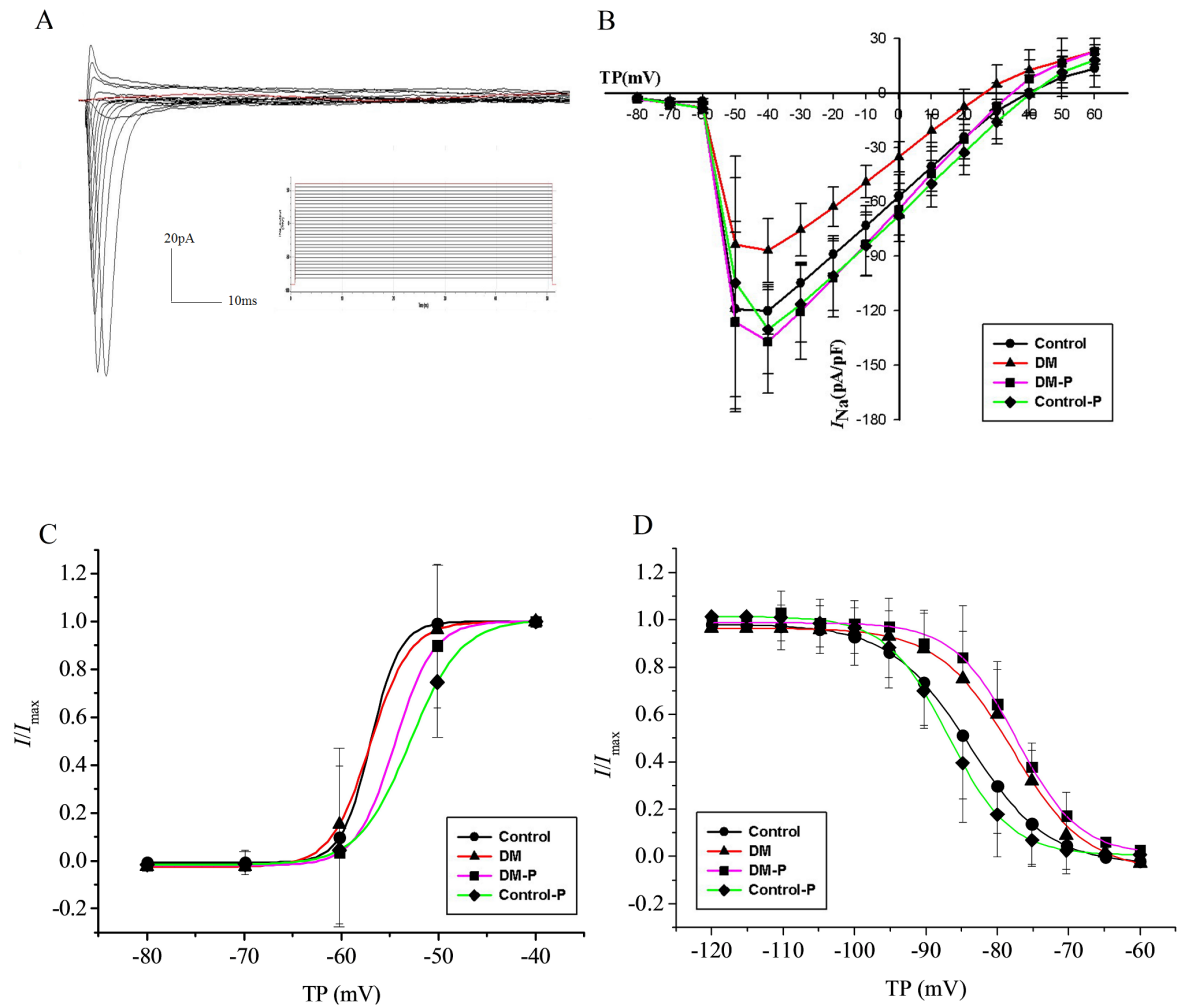
**A.** representative *I*_Na_ evoked by the applied protocol; **B.** *I*_Na_ densities and peak *I*_Na_ density as a function of test potential; **C.** The steady-state activation curve of *I*_Na;_ **D.** The steady-state inactivation curve of *I*_Na_, V1/2inact, TP: Test potential.

The average value of cell capacitance is 68.51±10.52 pF (*n* = 11) in the Control group, 68.91±14.23 pF (*n* = 10) in the DM group, 67.19±11.17 pF (*n* = 8) in the DM-P group, and 66.20±9.88 pF (*n* = 9) in the Control-P group. Figure 1(A) shows *I*_Na_ properties in response to the applied protocolFigure 1 (B) depicts *I*_Na_ densities and peak *I*_Na_ density as a function of test potential. Compared to the control group, the DM group did not exhibit differing morphology of the I-V curve, but demonstrated lower *I*_Na_ densities at all voltages (–60 mV to +35 mV) (*P* *<* 0.05), with a 72.29% reduction in *I*_Na_ density at –40 mV compared to the Control group. Peak *I*_Na_ density in the DM-P group increased by 157.92% compared with the DM group.

### Activation and inactivation properties of I_Na_ channels

The steady-state *I*_Na_ activation curve is shown in Figure 1(C) and the different activation and inactivation parameters are summarized in Table 2. *k* is the slope factor of the inactivation curve. The experimental data of the voltage dependent activation were fitted by Boltzmann equation. The membrane potential at which half-activation occurs, V1/2act, was not significantly different between the Control and DM groups (*P* > 0.05). It was not altered by probucol in either group (*P* > 0.05). The steady-state *I*_Na_ inactivation curve is shown in Figure 1(D). Together, the above data indicate that neither DM nor probucol modified the voltage-dependent activation or inactivation properties of *I*_Na_.

**Table 2:** The shifts in activation and inactivation variables of I_Na_ (*x*±s)

Group	Control	DM	DM-P	Control-P
*k*	1.49±0.37	2.03±0.69	1.98±0.28	2.63±0.34
V_1/2_ (mV)	-56.97±0.78	-57.01±1.12	-54.48±0.74	-52.93±0.48
V_1/2inact_(mV)	5.53±0.52	5.20±0.59	4.38±0.42	4.33±0.31
*k*_inact_	-84.00±0.57	-77.91±0.70	-77.32±0.49	-86.86±0.35

V1/2act (membrane voltage [Vm] at which half-activation occurs;V_1/2inact_: Vm at which half-inactivation occurs; *k*: the activation slope factor; *k*_inact_: inactivation slope factor

### I_CaL_ changes

**Figure 2:**
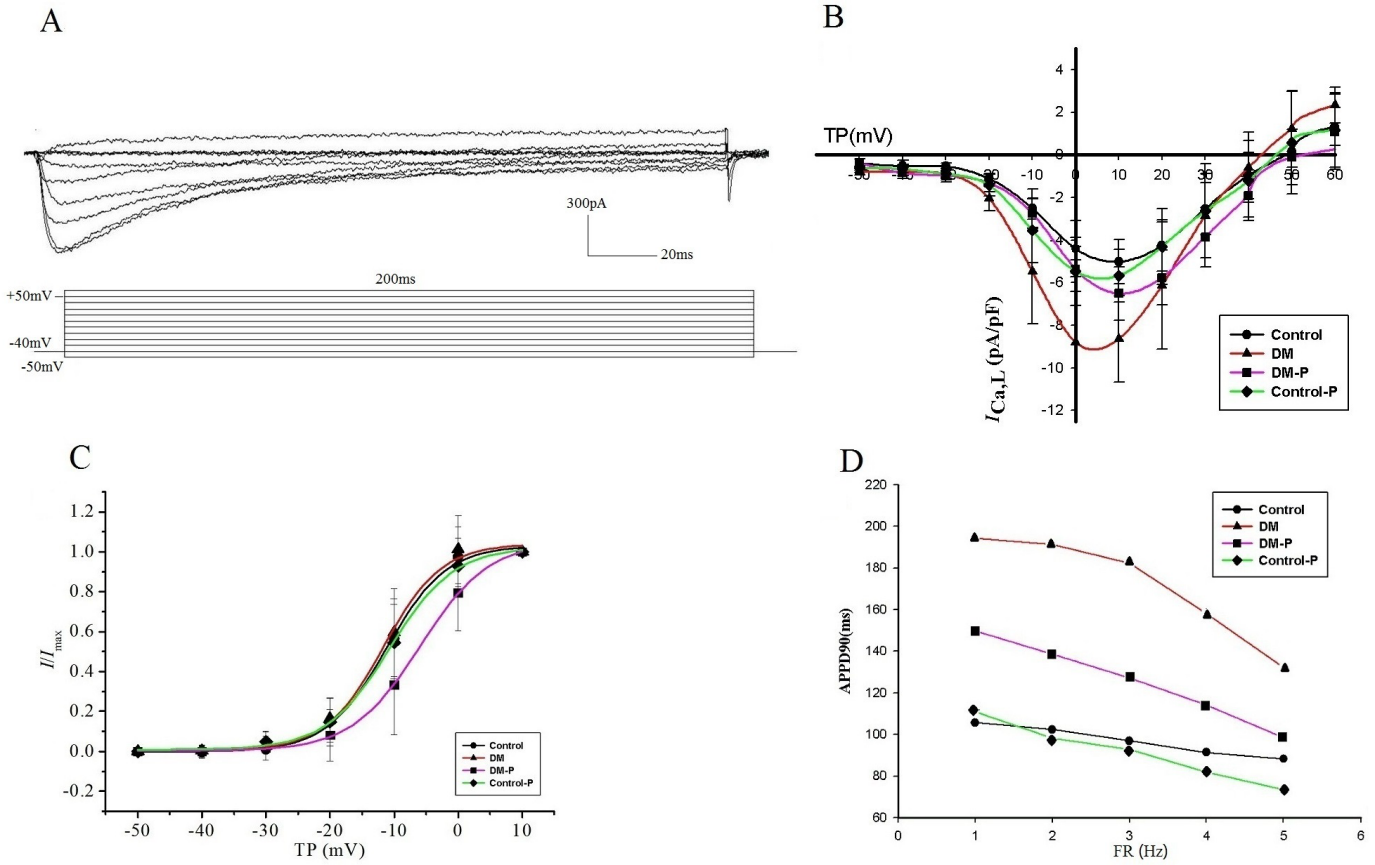
**A.** representative *I*_caL_ evoked by the applied protocol; **B.** *I*_caL_ densities and peak *I*_caL_ density as a function of test potential, TP: Test potential; **C.** The steady-state activation curve of IcaL. TP: Test potential; **D.** Rate-dependent APD90 changes.

The mean cell capacitance was 55.22±10.37pF (*n* = 12) in the Control group, 57.63±14.49 pF (*n* = 10) in the DM group, 57.79±14.38 pF (*n* = 10) in the DM-P group, and 59.57±9.24 pF (*n* = 13) in the Control-P group. Figure 2(A) shows *I*_caL_ properties in response to the applied protocol. Figure 2(B) illustrates *I*_caL_ densities and peak *I*_caL_ as a function of the test potential. Compared to the Control group, the DM group did not show differing morphology in the I-V curve, but had larger *I*_caL_ at all voltages steps tested between –60 mV to +35 mV (*P* *<* 0.05). Peak *I*_caL_ currents in the DM-P group were reduced by 62.68% compared to the DM group. The maximal *I*_caL_ amplitude was between 0 mV and 10 mV in all four experimental groups.

### Activation characteristics of I_CaL_ in isolated atrial myocytes

The steady-state *I_caL_* activation curve is shown in Figure 2 (C), in which the data were fitted by Boltzmann equation. V1/2act was not significantly different between the Control and DM groups (*P* > 0.05). Probucol increased V1/2act compared to the Control or DM groups (Control-11.21±0.51mv, DM-11.63±0.49mv, DM-P-5.91±0.71mv, Control-P-10.69±0.56mv, *P* < 0.05). A right shift in the activation curve was observed in the DM-P group, i.e. a given value of Vm the degree of current activation was smaller in the DM-P group. *k* in four groups are Control 4.67±0.49, DM 4.50±0.46, DM-P 5.37±0.57, Control-P 5.00±0.54, respectively, *P* > 0.05.

### Properties of the cellular action potential

The resting membrane potential was not altered in DM group with an average of −68.41±6.04 mV (*n* = 12 cells) in control rabbits compared with −67.70±6.20 mV (*n* = 12), −64.26±6.94 mV (*n* = 13), and −64.14±5.28 mV (*n* = 10) in DM, DM-P, and Control-P rabbits, respectively (*P* > 0.05). APD_90_ was shortened with increasing stimulation frequencies in all four experimental groups (Figure (2D)). Prolonged APD_90_ and APD_50_ values in the DM group were observed compared to the Control group; these changes were abolished by probucol (Figure 3, Table 3). Mean APD, AP amplitude and maximum upstroke velocity are displayed in DM did not significantly affect rate-dependent APD properties.

**Table 3:** Mean values of APD90 and APD50(*x*±s)

	Control (*n* = 12)	DM(*n* = 12)	DM-P (*n* = 13)	Control-P(*n* = 10)
RP(mv)	-78.41±6.04	-77.70±6.20	-74.26±6.94	-74.14±5.28
APD90-1Hz	105.69±29.77	194.34±45.47*	149.75±52.24#	110.93±23.37#
APD90-2Hz	102.34±26.39	191.19±42.04*	138.62±48.76#	98.08±30.55#
APD90-3Hz	96.92±25.01	182.40±40.93*	127.25±43.13#	92.95±30.41#
APD90-4Hz	91.38±23.33	158.28±16.09*	114.11±35.16#	81.95±26.03#
APD90-5Hz	88.27±22.2	132.21±11.62*	98.51±25.32#	73.62±25.11#
APD50-1Hz	35.26±20.34	69.65±24.02*	70.22±35.90*	44.50±11.53
APD50-2Hz	29.80±13.57	64.10±20.15*	57.71±25.89*	35.21±13.17
APD50-3Hz	27.97±8.14	182.40±40.93*	46.32±12.04#	30.26±10.76#
APD50-4Hz	24.50±6.23	63.05±28.62*	44.63±12.39	24.27±11.23
APD50-5Hz	20.51±5.15	55.83±28.40*	37.62±9.59#	22.06±10.68
AP-amplitude(mv)	204.96±20.14	134.54±24.58*	134.69±14.01*	188.85±14.79#
APA (mV)	204.96±20.14	134.54±24.58*	134.69±14.01*	188.85±14.79#
Vmax (V/s)	81.44±7.42	42.37±10.64*	52.23±9.39*#	82.31±7.74#

Abbreviations: RP: resting potential ;APA: action potential amplitude; Vmax: maximum rate of rise of the action potential; APD50, APD90: action potential duration at 50 and 90% repolarization;

* Compared with Control group *P* < 0.05;

# Compared with DM group *P* < 0.05

**Figure 3:**
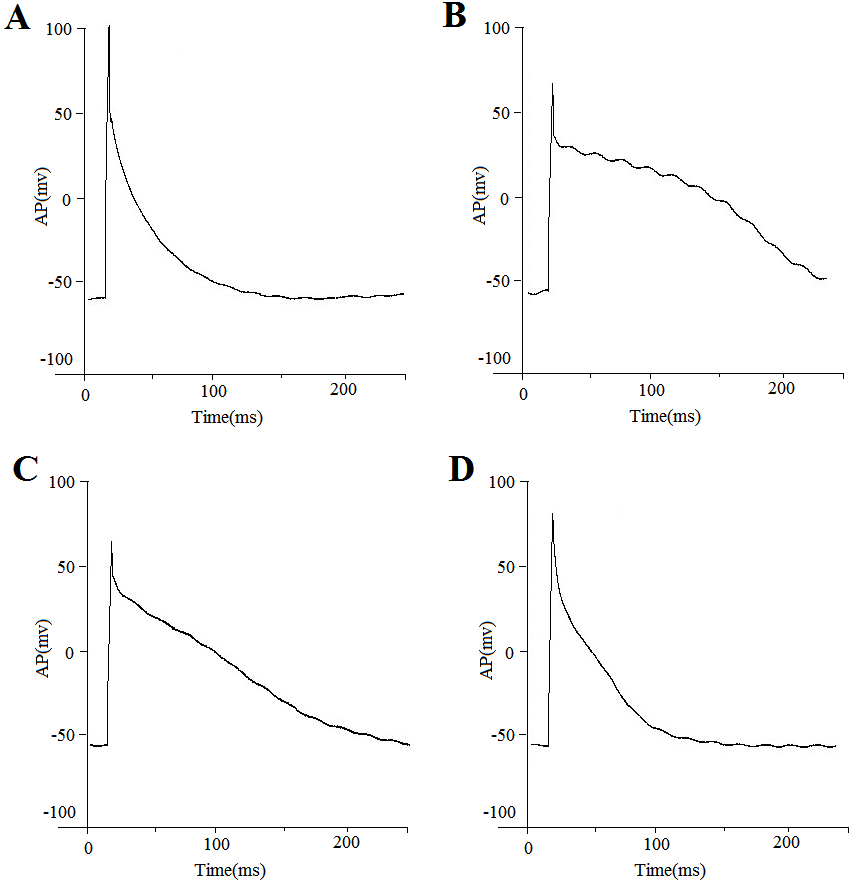
Action potential recordings from representative cells obtained from a **A.** control group; **B.** DM group; **C.** DM-P group and **D.** Control-P group. Action potentials were recorded in current-clamp mode at the frequencies indicated.

## DISCUSSION

In this study, we demonstrated: 1) diabetes reduced *I*_Na_ densities in atrial tissue, while leaving V1/2act or *k*act of *I*_Na _intact; 2) Whilst diabetes did not alter the morphology of the *I*_caL _I-V curve, it increased *I*_caL _densities. Probucol prevented the reduction of *I*_Na_ densities and the increase in *I*_caL _densities, increased V1/2act and produced a right shift in the activation curve, and *I*_caL_ was smaller at any given value of Vm compare with the DM group; 3) DM prolonged APD_90_ and APD_50_, and these changes were abolished by probucol.

### Effects of diabetes mellitus and probucol on I_Na_

Many isoforms of the voltage-gated Na^+^ channels have been described in many current literatures and the predominant isoform was found in cardiac tissue. It is known that Nav1.5 is encoded by SCN5A and *I*_Na_ activation contributes to the upstroke of cardiac AP [15]. Our previous experiments have shown that increased atrial interstitial fibrosis and higher vulnerability to AF was evident under hyperglycemic conditions in a DM rabbit model induced by alloxan [8]. These pathological changes were associated with higher expression levels of several proteins involved in inflammation, including transforming growth factor-β (TGF-β), TNF-α and NF-κB [7-9]. Moreover, prolonged APD_90_ and APD_50_ values in isolated left atrial cardiomyocytes from diabetic rabbit hearts were demonstrated, and these changes were associated with I_Na _downregulation and *I*_CaL_ upregulation [8]. We argue the ion channel remodeling can be explained by altered cellular signaling, such as protein kinase C (PKC) activation [16-17]. The activity of the cardiac Na^+^ channel can be regulated by post-translational modification such as phosphorylation events, through increased protein kinase A (PKA) and PKC [18]. This reduction in *I*_Na_ can be prevented by the antioxidant enzyme SOD or a PKC inhibitor. PKCα-mediated phosphorylation of the Na^+^ channel has been reported to result in immediate reduction of Nav1.5 channels [19]. Also, previous studies suggest that PKC decreases *I*_Na_, but this was not due to altered channel gating mechanisms [20]. Instead, this may involve alterations in transcriptional activity of the channel. In supporting of this notion, the promoter region of the SCN5a gene has a consensus NF-κB binding site [21]. Indeed, angiotensin II, which is elevated in cardiometabolic disorders, can lower Na^+^ channel expression mediated by higher levels of oxidative stress from H_2_O_2_. The latter can promote NF-κB binding to the Na^+^ channel promoter in turn leading to a decrease in Na^+^ channel expression in the plasma membrane [22]. Therefore, multiple interacting molecular mechanisms can alter the activity and expression of Na^+^ channels to mediate adverse electrophysiological remodeling.

Probucol is a potent antioxidant compound that has promising therapeutic effects in patients with DM in randomized clinical trials. It is an anti-hyperlipidemic agent that can reduce redox imbalances by reducing myocardial lipid peroxidation and increasing the antioxidant system [23, 24] and by downregulating a number of key pro-inflammatory mediators such as NF-κB, TGF-β, HSP70 and TNF-α in a diabetes rabbit model produced by alloxan [9].Therefore, it is likely that probucol exerts multiple effects on Na^+^ channel during pathophysiological conditions through reduction in NF-κB- and PKC-mediated oxidative stress.

### Effects of diabetes mellitus and probucol on Ica and action potential

Voltage-gated Ca^2+^ channels (VGCCs) modulated by cytosolic Ca^2+^ determine myocardial contractility and APDs in cardiac tissue. Normally, VGCCs are closed during electrical diastole and activated by membrane depolarization, and it contributes a significant amount of inward current during the plateau of the APs. This inward Ca^2+^ influx is responsible for inducing further release from the intracellular store, the sarcoplasmic reticulum, leading to contractile activation [25].

Previous reports found reduced expression of VGCCs and SERCA2 in AF patients [26-30]. However, the role of VGCCs in postoperative AF is less clear. Van Wagoner et al [31] observed that higher *I*_Ca,L_ in cardiomyocytes isolated from the right atria in postoperative AF patients compared to those without AF, but a different study found no significant difference in *I*_Ca,L_ or APD in these two groups [32]. We believe that these conflicting results are likely due to different baseline characteristics of the subjects involved and different methods of detecting AF [33]. In our study, prolongations in both APD_90_ and APD_50_ values were observed in left atrial cardiomyocytes isolated from diabetic rabbit hearts. These changes were linked to higher *I*_Ca,L_ densities. These findings are consistent with those by Van Wagoner et al [31]. Prophylactic treatment using calcium channel antagonists can reduce or prevent alterations in ion channel expression in the diabetic atria [34, 35]. Since calcium overload is an important mechanism in mediating electrical instability [36], we hypothesized that higher *I*_Ca_ in DM is responsible for this observation. AF can be caused by triggered activity or by re-entry [37-39]. EADs develop in the context of APD prolongation, when inward currents (*I*_ca.L_ or late Na^+^ current (*I*_NaL_)) are increased, or when outward currents (I_K+_) decreased [46, 47]. The causal relationship between abnormal Ca^2+^ handling and AF development is bidirectional [32]. Thus, hydrogen peroxide (H_2_O_2_), a reactive oxygen species, can alter molecular signaling mechanisms that ultimately lead to altered cellular calcium homeostasis [47]. The data generated from our group demonstrate that through a reduction in oxidative stress, as shown using the anti-oxidant compound probucol, can prevent or reverse electrophysiological remodeling in the atria [9].

## MATERIALS AND METHODS

This study was approved by the Experimental Animal Administration Committee of Tianjin Medical University and Tianjin Municipal Commission for Experimental Animal Control, which follow the guidelines established by the U.S. National Institutes of Health.

### Experimental animals

Forty Japanese rabbits, weighted ~1.5–2.0 kg at the beginning of the study (Beijing Medical Animals Research Institute, Beijing, China), were randomly assigned to control group (Control, *n* = 10), alloxan-induced diabetic group (DM, *n* = 10), probucol-treated group (Control-P, *n* = 10), and probucol-treated diabetic group (DM-P, *n* = 10). In the DM and DM-P groups, alloxan monohydrate (Sigma ldrich Chemical, Saint Louis, MO, USA) was dissolved in sterile sodium chloride to achieve a concentration of 5% (W/V) and 120 mg/kg, and then was immediately administered into the marginal ear vein. The diabetic state was confirmed 48 hours later by measuring blood glucose levels of ≥14mmol/L. Rabbits in the DM-P and Control-P groups received probucol (Lunan Pharmaceutical Company, Shandong, China) orally (1,000 mg/day) for 8 weeks. The animals in the four groups were housed in cages on a standard laboratory pellet diet during the 8 week experimental period.

### Serum biochemical and oxidative stress parameters

Serum cholesterol, triglyceride, low density lipoprotein cholesterin (LDL-c), high density lipoprotein cholesterol (HDL-c), fasting insulin blood urea nitrogen (BUN), and creatinine (Cr) levels were measured using automated techniques. Antioxidant enzyme activities levels in serum and left atrial tissue, including superoxide dismutase (SOD), catalase (CAT), myeloperoxidase (MPO) and malondialdehyde (MDA), were detected using antioxidant enzyme activities kits (Nanjing Jianchen Bioengineering Institute, Jiangsu province, China).

### Single-cell electrophysiology

#### Cell isolation

The left atrium (LA) was used for isolation of single cardiomyocytes. The procedure for isolating cells was followed from a previously developed method [13]. A rabbit was anesthetized with 3% Pelltobarbitalum Natricum (30 mg/kg), and then the rabbit's heart was quickly removed from the torso and was placed in cold perfusion fluid (4 °C). The aorta was cannulated and connected to a Langendorff perfusion system filled with warmed (37 ±0.5 °C) Ca^2+^-free Tyrode's solution. Next, the heart was perfused at 30ml/min for 20 minutes, followed by a 20-minute perfusion containing collagenase (0.075%, CLS II, Worthington Biochemical, Lakewood, CO, USA) and 0.2% bovine serum albumin (BSA; Sigma Chemical Co., St. Louis, MO, USA). Cardiac tissue became opaque and mottled in appearance. Tissue from a well-perfused region of the LA free wall was removed using forceps, gently triturated, and maintained at room temperature in a high-K^+^ storage solution (KB solution). A small aliquot of the solution containing isolated cells was placed in a 2-mL chamber mounted on the stage of an inverted microscope (IX-50, Olympus Co., Tokyo, Japan). Five minutes following cell adhesion to the bottom of the chamber, cells were superfused with Tyrode's solution (3 mL/min) for 5 minutes, followed by extracellular solution for the recording of *I*_Na_ or *I*_caL_ at the same velocity and time of flow. Recordings of *I*_Na_ and *I*_caL_ were obtained 5 minutes following membrane rupture.

### Data acquisition

Membrane currents were measured at room temperature (22±1^o^C) using the whole-cell configuration of the patch-clamp technique [14]. Briefly, whole-cell configuration was made in Tyrode's solution. Pipette resistances were 2-4 mV. After achieving a gigaseal, the test-pulse current was nulled by adjusting the pipette capacitance compensator with both fast and slow components. After break-in, the whole-cell charging transient was nulled by adjusting whole cell capacitance and series resistance. Voltage control protocols were generated with Axopatch 200B amplifier/Digidata 1200B acquisition system using pCLAMP-10 software (Molecular Devices/Axon, Sunnyvale,CA). Whole-cell recording was analyzed using Clampfit 10.2. *I*_Na_, *I*_CaL_ and action potentials (APs) were recorded and filtered at 0.5 kHz using a low pass filter. The sampling frequency was set at 0.5 Hz for recording *I*_Na_ and 0.2 Hz for *I*_CaL_. The current-voltage relation was determined in the extracellular solution over a voltage range of -80 to +60 mV increased in 10-mV steps from a hold potential (HP) of -90mV for *I*_Na_ and -40 to +60 mV increased in 10-mV steps from a HP of -50mV for *I*_CaL_. The APs were recorded at 60, 120, 180, 240 and 300 bpm. APD was stabilized within 5 action potentials at each cycle length, and was measured at 50% (APD_50_) and 90% (APD_90_) of full repolarization.

### Statistical analysis

All results are presented as mean ± standard deviation. Comparisons between the groups were analyzed for statistical significance using the one-way analysis of variance (ANOVA) and Fisher's exact test, respectively. Differences with *P* < 0.05 were considered statistically significant. In order to ensure data validity from voltage clamp studies, similar numbers of cells from different regions of each heart were studied utilizing the same protocol (i.e., cells were distributed evenly across rabbits).
